# P-927. Clinical Outcome of Ampicillin plus Ceftriaxone Compared to Nafcillin or Cefazolin plus Gentamicin as an Empirical Treatment for Infective Endocarditis

**DOI:** 10.1093/ofid/ofae631.1118

**Published:** 2025-01-29

**Authors:** Min Seo Kang, Ji-won Hwang, Seung Woo Park, Kyong Ran Peck, Jihoon Kim, Eun Kyoung Kim, Sung-Ji Park, Jinyoung Yang, Sun Young Cho, Jae-Hoon Ko, Cheol-In Kang, Doo Ryeon Chung, Kyungmin Huh

**Affiliations:** Samsung Medical Center, Seoul, Seoul-t'ukpyolsi, Republic of Korea; Ilsan Paik Hospital, Goyang, Kyonggi-do, Republic of Korea; Samsung Medical Center, Seoul, Seoul-t'ukpyolsi, Republic of Korea; Samsung Medical Center, Seoul, Seoul-t'ukpyolsi, Republic of Korea; Samsung Medical Center, Seoul, Seoul-t'ukpyolsi, Republic of Korea; Samsung Medical Center, Seoul, Seoul-t'ukpyolsi, Republic of Korea; Samsung Medical Center, Seoul, Seoul-t'ukpyolsi, Republic of Korea; Samsung Medical Center, Seoul, Seoul-t'ukpyolsi, Republic of Korea; Samsung Medical Center, Seoul, Korea, Seoul, Seoul-t'ukpyolsi, Republic of Korea; Samsung Medical Center, Seoul, Seoul-t'ukpyolsi, Republic of Korea; Samsung Medical Center, Seoul, Seoul-t'ukpyolsi, Republic of Korea; samsung medical center, Seoul, Seoul-t'ukpyolsi, Republic of Korea; samsung medical center, Seoul, Seoul-t'ukpyolsi, Republic of Korea

## Abstract

**Background:**

A combination of anti-staphylococcal penicillin or cefazolin and gentamicin has been recommended as an empirical treatment regimen for native valve or late prosthetic valve infective endocarditis (NV/LPVIE), while the recent European Society of Cardiology Guidelines proposed ampicillin plus ceftriaxone (AMP+CRO) as an empirical option. However, clinical data supporting either regimen are scarce.

**Figure:**
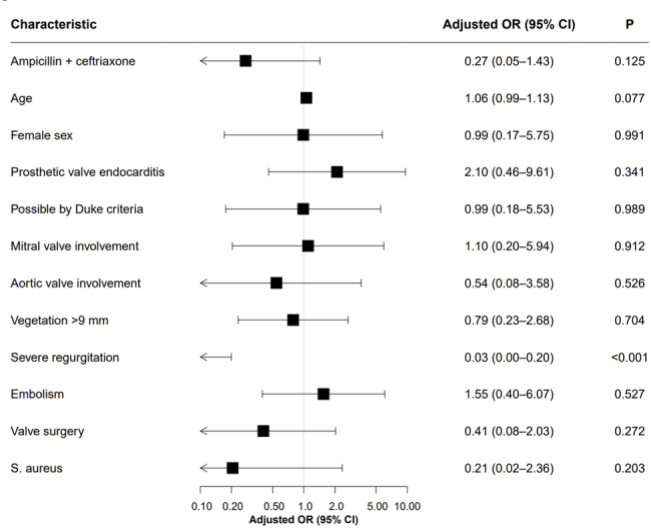

Risk factors of composite outcome in a Cox proportional hazard model with overlap weighting.

**Methods:**

A retrospective cohort study was conducted in a large tertiary care hospital. Adults aged ≥18 years who were diagnosed with NV/LPVIE by the modified Duke criteria from 2000 through 2022 were included. Patients were grouped by empirical antibiotic regimens including AMP+CRO versus nafcillin or cefazolin plus gentamicin (NAF/CFZ+GEN). Multiple logistic regression with overlap weighting was used to compare outcome between the two groups. Primary outcome was all-cause mortality or relapse within 6 months since diagnosis.

**Figure:**
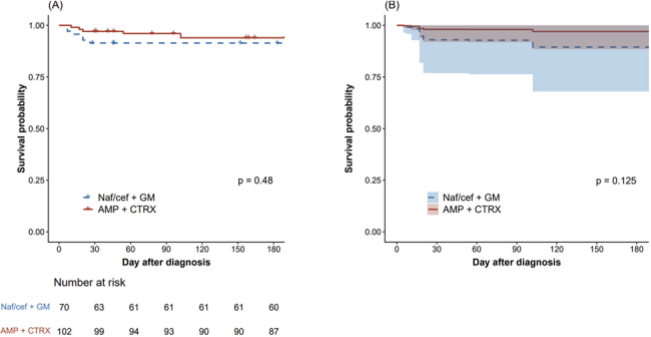

Survival probabilities by empirical treatment regimen. (A) Observed survival without relapse, (B) probability of survival without relapse in the Cox proportional hazard model with overlap weighting.

**Results:**

A total of 172 patients were included and divided into two groups by the empirical antibiotic regimens of AMP+CRO (n=102) or NAF/CFZ+GEN (n=70). Both groups exhibited similar baseline characteristics, including underlying diseases, preexisting valvular conditions, and involved valves. However, diabetes, cardiovascular diseases, and congestive heart failure were more common in the AMP+CRP group. Viridans group *Streptococcus* was the most frequently identified pathogen (33.3%) who were administered AMP+CRP, while *Staphylococcus aureus* was the most prevalent pathogen in the NAF/CFZ+GEN group (42.9%). In the AMP+CRO group, 6 (5.9%) met the composite outcome compared with 6 (8.6%) in the NAF/CFZ+GEN group (*P* = 0.551). There was also no statistically significant difference in the risk of composite outcome between the two groups in a multivariable analysis with overlap weighting (adjusted odds ratio, 0.27; 95% confidence interval, 0.05–1.43; *P* = 0.125).

**Figure:**
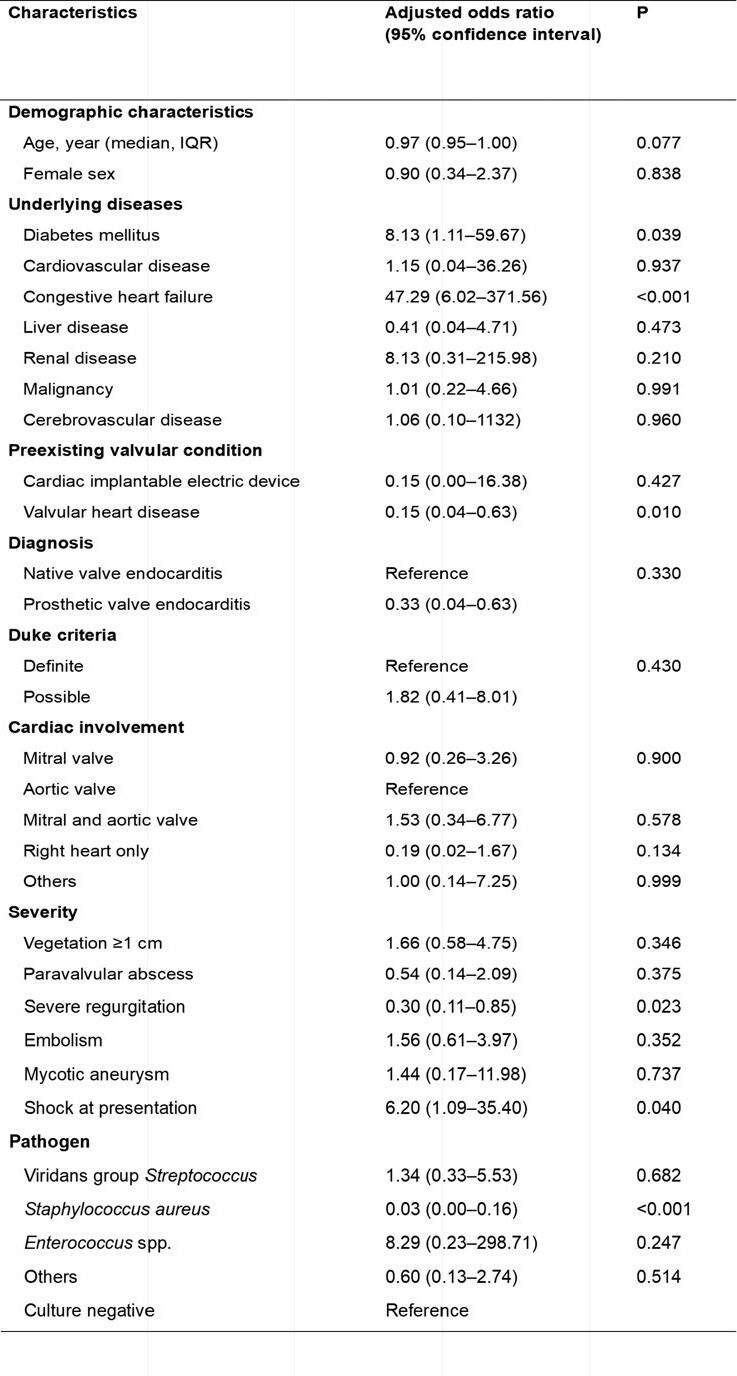

Factors associated with initial treatment with ampicillin + ceftriaxone in a logistic regression model

**Conclusion:**

In the patients with NV/LPVIE, AMP+CRO as empirical antibiotic therapy showed no significant difference in the all-cause mortality or relapse within 6 months since diagnosis compared to NAF/CFZ+GEN. Our results suggest that AMP+CRO can be used as a primary empirical regimen for the treatment of NV/LPVIE.

**Figure:**
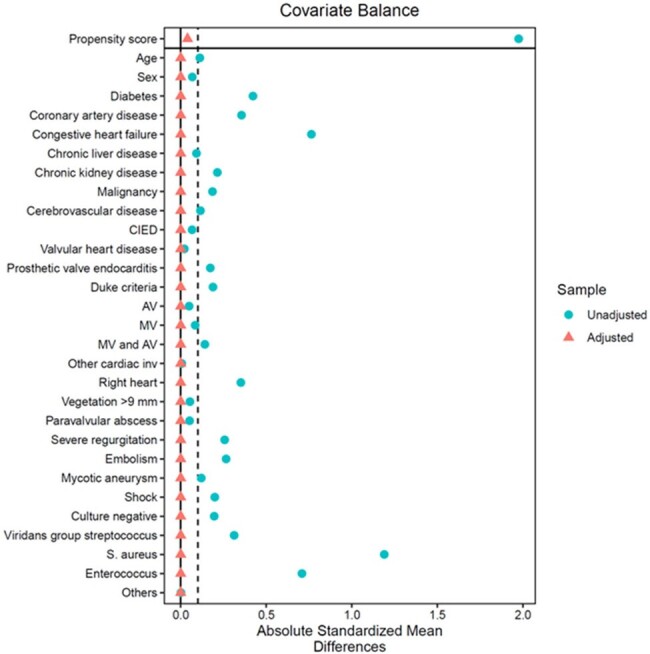

Covariate balance before and after overlap weighting

**Disclosures:**

**All Authors**: No reported disclosures

